# The everchanging framework of autoinflammation

**DOI:** 10.1007/s11739-021-02751-7

**Published:** 2021-05-17

**Authors:** Raffaele Manna, Donato Rigante

**Affiliations:** 1 Department of Internal Medicine, Fondazione Policlinico A. Gemelli IRCCS, Rare Diseases and Periodic Fevers Research Centre, Università Cattolica Sacro Cuore, Largo A. Gemelli no. 8, 00168 Rome, Italy; 2 Rare Diseases and Periodic Fevers Research Centre, Università Cattolica Sacro Cuore, Rome, Italy; 3 Department of Life Sciences and Public Health, Fondazione Policlinico A. Gemelli IRCCS, Rome, Italy

**Keywords:** Autoinflammatory disease, Autoinflammation, Recurrent fever, Inflammasome, Inflammasomopathy, Interleukin-1, Nuclear factor-κB, Interferon, Innovative biotechnologies, Personalized medicine, Child

## Abstract

The innate immunity works as a defence bullwark that safeguards healthy tissues with the power of detecting infectious agents in the human body: errors in the context of innate immunity identify autoinflammatory disorders (AIDs), which arise as bouts of aberrant inflammation with little or no involvement of T and B cells and neither recognized infections, nor associated autoimmune phenomena. Hereditary AIDs tend to have a pediatric-onset heralded by stereotyped inflammatory symptoms and fever, while AIDs without an ascertained cause, such as systemic juvenile idiopathic arthritis, derive from the interaction of genetic factors with environmental noxae and are unevenly defined. A dysregulated inflammasome activation promotes the best-known family of AIDs, as well as several degenerative and metabolic disorders, but also nuclear factor κB- and interferon-mediated conditions have been framed as AIDs: the zenith of inflammatory flares marks different phenotypes, but diagnosis may go unnoticed until adulthood due to downplayed symptoms and complex kaleidoscopic presentations. This review summarizes the main AIDs encountered in childhood with special emphasis on the clinical stigmata that may help establish a correct framework and blueprints to empower young scientists in the recognition of AIDs. The description focuses inflammasomopathies as paradigms of interleukinopathies, nuclear factor-κB -related disorders and interferonopathies. The challenges in the management of AIDs during childhood have been recently boosted by numerous therapeutic options derived from genomically-based approaches, which have led to identify targeted biologic agents as rationalized treatments and achieve more tangible perspectives of disease control.

**Supplementary Information:**

The online version contains supplementary material available at 10.1007/s11739-021-02751-7.

## Introduction

**Fig. 1:**
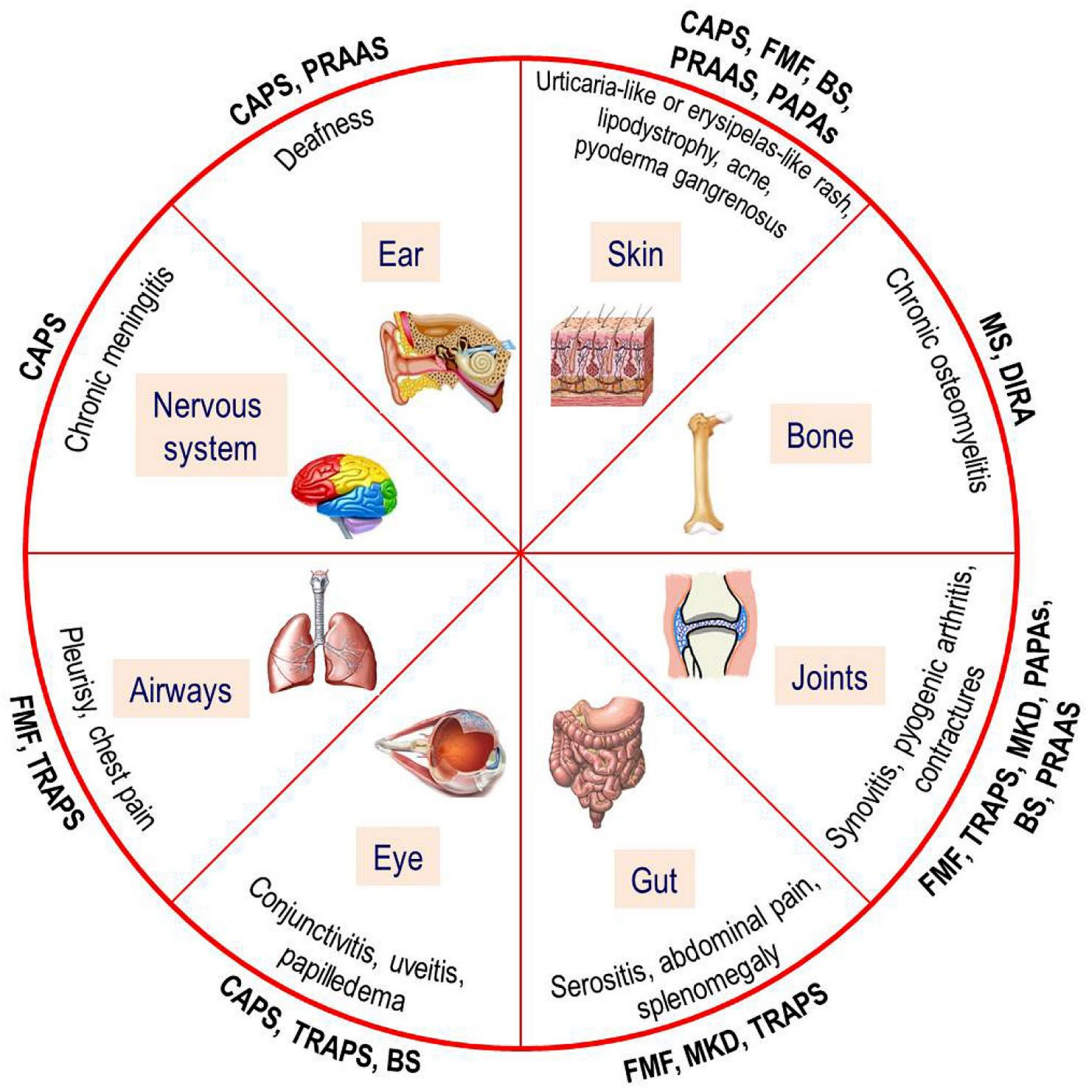
Pie chart depicting the organs involved and general clinical signs related to the hereditary monogenic autoinflammatory disorders

Inflammation is a vital physiological response triggered by protean noxious agents in all metazoan organisms, and the term “autoinflammation” specifically refers to innate immunity-mediated inflammation. Autoinflammatory disorders (AIDs) represent a heterogeneous group of diseases with little or no involvement of T and B cells, caused by primary dysregulated processing of innate immunity and oversecretion of proinflammatory cytokines: multiple genetic defects can be related to the structure of inflammasomes or proteasome as well as cytokine receptors and regulator molecules, with different enzymes involved in their function and activation, but all are characterized by recurrent fevers and recurrent inflammatory manifestations [1]. Pediatricians are frequently called to evaluate children with a history of periodic fevers and their first task is differentiating infectious and non-infectious causes of fever, taking into account immunodeficiencies, autoimmune disorders and even neoplastic diseases. A minority of these children might conceal abnormalities of the innate immune system like AIDs, characterized by the fluctuation of febrile inflammatory signs affecting skin, joints, bone tissue, gut, airways, eyes, ears or nervous system (as depicted in Fig. 1) with a variable modality of recurrence, alternating with periods of complete wellness [2]. The description of tardive manifestations, veiled phenotypes and atypical clinical signs beginning in adulthood has been more and more reported in recent times for different AIDs, requiring that many specialists become confident with concepts, details and management of autoinflammation [3].

The participation of different inflammasomes, i.e. cytosolic protein complexes acting as sensors of cellular homeostasis, into the pathogenesis of many AIDs has been confirmed by a host of studies, and since the discovery of inflammasome in 2002 there has been burgeoning recognition of inflammasome complexity and important functions. In general terms, an “active” inflammasome consists of a central scaffold protein, for which it is named (like NLRP1, NLRP3, NLRP6, NLRC4, AIM2), an adaptor apoptosis-associated speck-like protein (ASC) containing a caspase activation and recruitment domain (CARD), which is mandatory, and the precursor form of the caspase-1 enzyme, named pro-caspase-1 [4]. Pyrin is distinct among inflammasome‐forming receptors such as NLRP1, NLRP3 or AIM2, and a peculiar pyrin inflammasome, that comprises ASC and caspase-1, has evolved as an innate immune sensor to detect bacterial toxin-induced Rho guanosine triphosphatase (Rho GTPase) inactivation. A dysregulation of inflammasome activity is associated with a number of AIDs, but how inflammasomes are activated is still undeciphered. In the case of cryopyrin (also named NLRP3, meaning NACHT, LRR and PYD domains-containing protein 3), one of the most studied caspase-1 activators, several signals related to cell damage and stress are claimed to activate a specific NLRP3-inflammasome, which finally leads to cleavage of the pro-interleukin (IL)-1β into its active form through caspase-1 intervention [5]. Indeed, the NLRP3-inflammasome is one of the main sources of IL-1β and acts also in several inflammatory disorders, including type 2 diabetes, gout and atherosclerosis [6]. The regulation of inflammasome activity and many therapeutic interventions targeting molecules related to inflammasomes or directly IL-1 signaling constitute spurring areas of basic and translational research. Indeed, IL-1 has been depicted as an "Ariadne's thread" through the labyrinth of AIDs and even autoimmune disorders, emphasizing the blurred boundary between both innate and adaptive immune systems [7]. The effectiveness of IL-1 blockade in treating many AIDs has also paved the way to targeting IL-1 in other non-hereditary conditions, such as systemic juvenile idiopathic arthritis, periodic fever/aphthosis/pharyngitis/adenitis syndrome and Kawasaki disease. The family of AIDs is continuously expanding and actually includes “classic” inflammasomopathies, nuclear factor (NF)- κB -mediated disorders (also named “relopathies”) and interferonopathies. This review focuses on the most recent advances that have helped to discriminate a potential genetic cause of recurrent febrile inflammatory episodes in children and elucidate a streamlined classification of hereditary monogenic AIDs combined with clues evoking multifactorial non-hereditary AIDs.

## The inflammasomopathies

Inflammasomes are intracellular protein complexes with a critical role in host defense against infections and non-infectious events through the proteolytic maturation of proinflammatory cytokines: the signaling process initiates with the detection of endogenous and/or external danger signals by specific sensors, followed by the polymerization from the sensor to downstream adaptor and then to the effector, caspase-1. An abnormal activation of the inflammasome characterizes all “inflammasomopathies”. These rare diseases are caused by mutations in genes encoding recognition receptors, upstream or downstream signaling molecules and sensor proteins involved in many innate immunity pathways [1]. In general terms, each typical febrile attack of children with inflammasomopathies is self-limited, lasts from a few days to some weeks, is followed by spontaneous resolution of every clinical manifestation and is separated by variable symptom-free intervals [8].

**Fig. 2:**
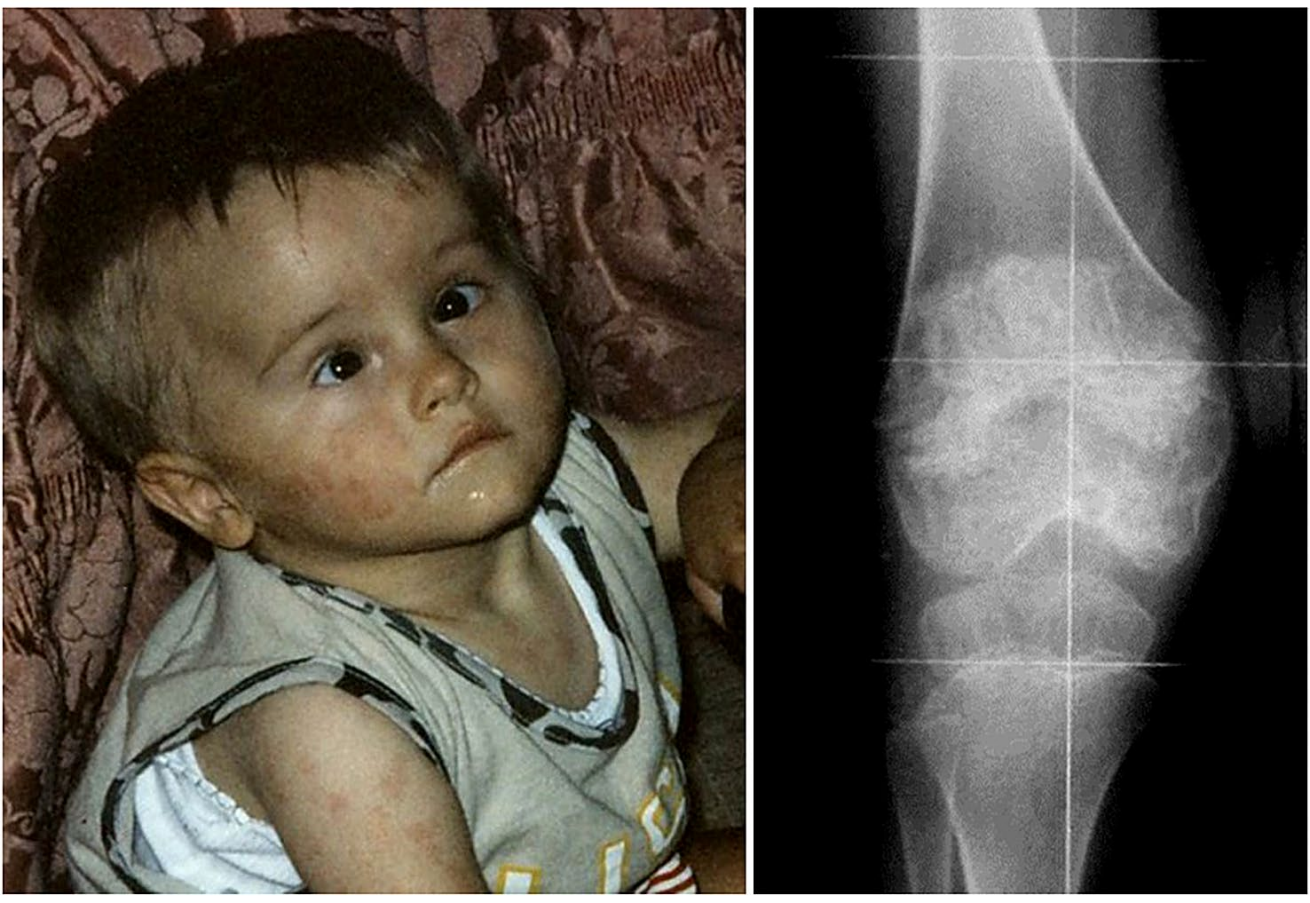
A peculiar dysmorphic face with prominent forehead, saddle nose and midface hypoplasia characterizes CINCA syndrome in combination with chronic urticaria-like rash (patient’s parents gave their formal informed consent for this publication); hypertrophic osteopathy involving distal femura and patellae in the same child (on the right side)

A subverted inflammasome homeostasis leading to IL-1 oversecretion characterizes the cryopyrin-associated periodic syndrome (CAPS), with three clinical sceneries of increasing severity which usually start in the first infancy [9]: familial cold-induced autoinflammatory syndrome (FCAS), Muckle-Wells syndrome (MWS) and chronic infantile neurologic cutaneous articular syndrome (or CINCA syndrome, also referred as “neonatal onset multi-system inflammatory disorder”), all caused by dominant missense mutations in the NACHT domain of the same *NLRP3* gene encoding the cryopyrin protein. *NLRP3* mutations have a gain-of-function power and lead to mutant cryopyrins exhibiting a constitutive activation of the NLRP3-inflammasome, which enhance IL-1 production [10]. CAPS phenotypes display nonspecific, but unique clinical signs: dermatologic, musculo-skeletal, ocular, otologic and neurologic symptoms combined with chronic systemic inflammation are highly characteristic (a summary of these general clinical signs is reported in Table 1, Supplementary material). In particular, the manifestations of FCAS and MWS show a significant overlap in childhood, including recurrent fevers, nonpruritic migratory urticaria-like rash mostly induced by cold exposure, conjunctivitis, arthralgia and fatigue. Conversely, CINCA syndrome is depicted by neonatal onset of the same signs combined with hypertrophic osteopathy involving distal femura and patellae, chronic aseptic meningitis with papilledema, sensorineural hearing loss and peculiar dysmorphic face (Fig. 2) [11]. Making a diagnosis of CAPS is challenging, as several patients show a variable mix of multi-system symptoms with heterogeneous disease courses. Genetic analysis can corroborate the clinical suspicion of CAPS, but around 40% of patients might not carry a specific *NLRP3* mutation at the conventional Sanger sequencing test, while the application of novel more sensitive genetic methods like next-generation sequencing can disclose somatic mosaicism, consistent with specific *NLRP3* mutations occurred during embryogenesis [12]. Immunosuppressant and biological medications had been used prior to the elucidation of CAPS-specific IL-1 signaling, though with poor clinical responses [13]; conversely, biologicals that target IL-1 were introduced along with the discovery of cryopyrin role in activating caspase-1, and indeed IL-1 antagonism is profoundly effective in most inflammasomopathies caused by mutations in genes related to innate immunity sensors with IL-1 overactivity. The recombinant human IL-1 receptor antagonist anakinra has been the first biologic designed for the selective blockade of IL-1, improving different complications of CAPS chronic inflammation and even stabilizing CAPS neurological signs [14]. Long-term IL-1 blockade has also shown striking effects on the CINCA skeletal dysplasia [15, 16]. Both the human monoclonal antibody canakinumab targeting IL-1β and the dimeric fusion protein rilonacept that neutralizes IL-1β are extremely effective and have FDA approval for CAPS treatment [17].

**Fig. 3:**
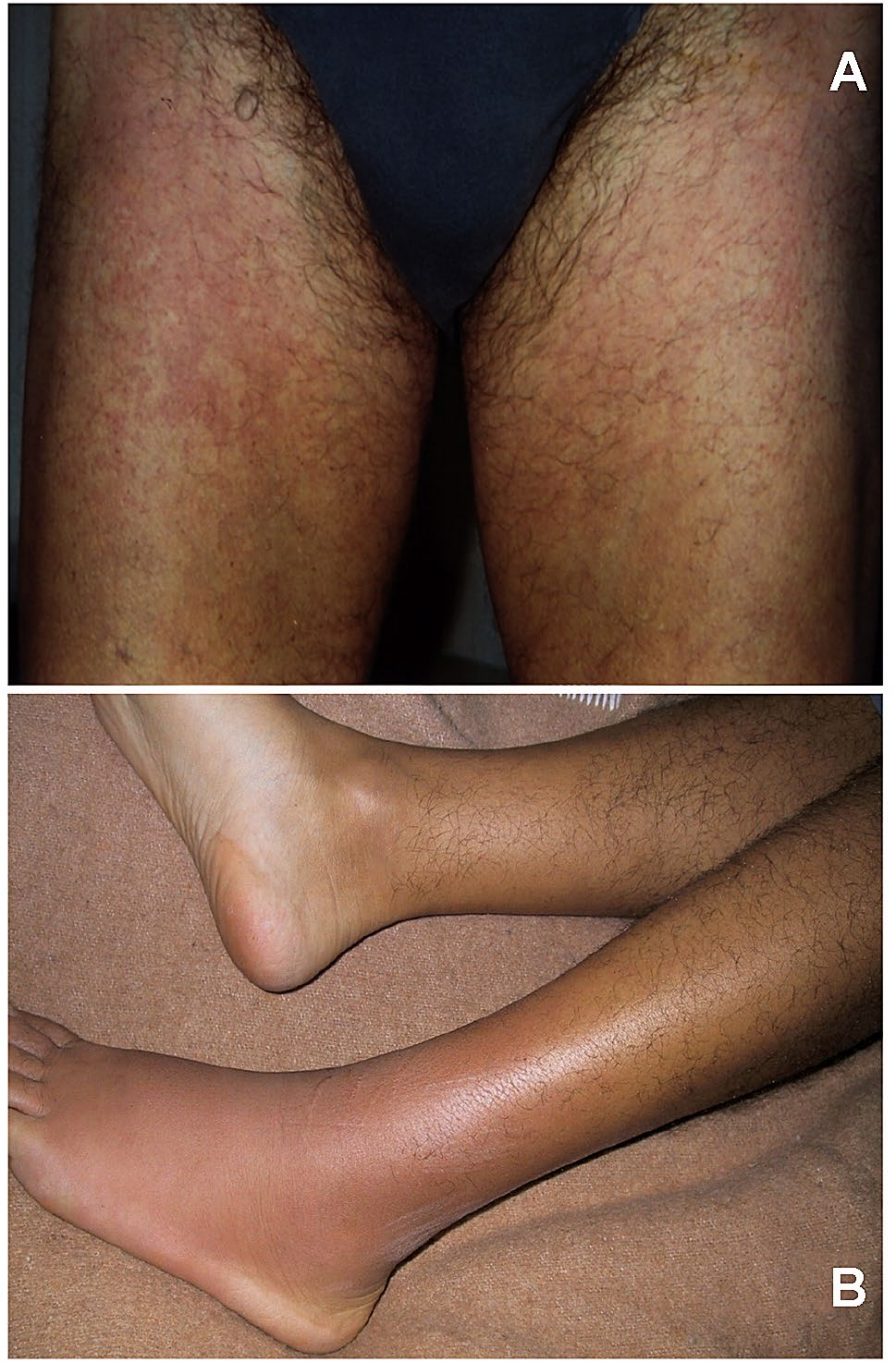
Centrifugal migratory erythematous skin lesions characteristic of febrile flares occurring in the autosomal dominant familial periodic fever (**a**) and a typical short-lived erysipelas-like rash during a febrile attack of familial Mediterranean fever (**b**)

The autosomal dominant familial periodic fever, best known as tumor necrosis factor (TNF) receptor-associated periodic syndrome (or TRAPS), initially named “familial Hibernian fever”, is the most prevalent dominantly inherited disease among inflammasomopathies, caused by monoallelic missense mutations in the *TNFRSF1A* gene encoding the 55kD receptor of TNF (TNFR) [18]. Many mechanisms have been reported to explain the recurrent febrile attacks of these patients, which may recur a few times per year and have a longer duration (if compared with other AIDs): mutations which result in abnormally folded TNFRs give rise to receptors retained in the endoplasmic reticulum, which do not progress to work at the cell surface [19]. The age of TRAPS onset spans from the first infancy to adulthood, and centrifugal migratory erythematous skin lesions (Fig. 3), myalgia with muscle edema, arthralgia, abdominal pain, periorbital edema or painful conjunctivitis are quite typical manifestations; additionally, TRAPS prognosis is determined by the occurrence of renal amyloidosis [20]. Idiopathic recurrent acute pericarditis, sometimes as isolated manifestation, is the most reported cardiovascular abnormality in these patients [21]. Treatment with corticosteroids alleviate most inflammatory symptoms without affecting the frequency of attacks. The clinical response to corticosteroids usually helps to differentiate TRAPS, but the exact diagnosis requires genetic analysis with the demonstration of a *TNFRSF1A* mutation [22]. Anti-TNF treatment has been experimented due to the observation that TRAPS molecular defect was sometimes associated with impaired TNFR shedding from cell membranes, but its effect was contradictory [23]. Conversely, the IL-1 targeted treatment with blockers of IL-1 action, such as anakinra and canakinumab, has reached impressive and enduring clinical results [24].

A recessively inherited inflammosomopathy is mevalonate kinase deficiency (MKD), which causes attacks of variable severity ranging from the milder hyper-IgD syndrome (HIDS) to the most severe mevalonic aciduria (MA), considered as a continuous disease spectrum caused by mutations in the *MVK* gene. As a consequence of mutations, there is the poor or absent enzymatic activity of mevalonate kinase, the peroxysomal enzyme involved in the catabolism of mevalonic acid, which leads to a shortage of bioactive isoprenoids, perturbation of different cell signals and defective autophagy; in particular, impaired geranylgeranylation of Rho GTPases leads to increased pyrin activity and subsequent hyperproduction of IL-1 [25]. The disease starts in toddlers with self-limited flares characterized by fever, cervical lymph node enlargement, severe abdominal pain with diarrhoea or vomiting, arthralgia and heterogeneous rashes; the first attack may be frequently triggered by immunizations [26]. Macrophage activation syndrome has been reported concurrently with inflammatory flares of MKD [27]. A polyclonal increase of serum IgD with values exceeding 100 IU/ml can be found in 80% of children with MKD, while more striking is enhanced urinary excretion of mevalonic acid during febrile attacks [28]. MA is the most severe expression of MKD, caused by the absolute deficiency of mevalonate kinase, leading to psychomotor retardation, microcephaly, ataxia, dysmorphic features, cholestatic liver disease and recurrent febrile attacks similar to HIDS [29]. Treatment of MKD is feasible with corticosteroids and anti-inflammatory drugs, but the most brilliant results have been obtained with both IL-1 receptor antagonist anakinra (given daily or “on demand”) and human anti-IL-1β monoclonal antibody canakinumab. In particular, canakinumab is the only well-studied and most effective treatment for MKD, with 35% of patients reaching a complete remission [30].

The oldest disease belonging to the group of inflammasomopathies and the most prevalent among all hereditary AIDs worldwide is familial Mediterranean fever (FMF), a recessively inherited disease characterized by episodic fevers with some combination of self-limited sterile peritonitis, pleurisy, transient arthritis of large joints or a characteristic short-lived ankle rash (Fig. 3). The FMF-involved gene is *MEFV*, which encodes pyrin (also named TRIM20 or marenostrin), a cytoplasmic and nuclear protein also involved in the pathogenesis of MKD and other inflammasomopathies, which can sense pathogen-induced modifications of host Rho GTPases. In particular, pyrin recognizes Rho-modifying bacterial toxins which inactivate RhoA GTPase and dephosphorylate pyrin: GTPase-mediated pyrin phosphorylation inhibits IL-1 production, while mutant pyrins cannot be phosphorylated and activate the pyrin inflammasome, increasing both production and release of IL-1 [31]. Carriers of *MEFV* mutations are particularly concentrated in the areas of the Mediterranean basin [32], though the highest prevalence of healthy carriers is reported in the Armenian people, suggesting a selective advantage of these genotypes, probably to an endemic pathogen. However, the identification of biallelic *MEFV* variants is required to confirm FMF diagnosis at a genetic level. Genetic analysis can be sometimes inconclusive even in individuals with a suggestive FMF picture, who might show only one *MEFV* variant: approximately 30% of all cases clinically diagnosed with FMF carry only one demonstrable mutation, despite an extensive search for a second disease-causing variant. In such cases, a 6-month trial with colchicine can help establishing the diagnosis [33]. A recurrent pericarditis might be part of the clinical spectrum of FMF [34]. The most severe complication affecting the prognosis of FMF patients is amyloidosis that can lead to dysfunction of vital organs, such as kidneys, and amyloid-A nephropathy can usually progress to end-stage renal disease, which is dependent on both specific *MEFV* mutations and other less defined environmental factors [35]. Clinical diagnosis of FMF can be established via both Tel-Hashomer and Livneh’s criteria [36, 37] (which have been listed in Table 2, Supplementary material).

In 1972 the serendipitous discovery that colchicine, an inhibitor of microtubule polymerization, was effective in the prevention of FMF attacks revolutionized the management of these patients: in fact, at an adequate dose and on full compliance, colchicine prevents FMF attacks and even occurrence of renal AA amyloidosis in approximately 95% of patients. It is also known that colchicine undergoes renal clearance, but sometimes this drug is poorly tolerated from the gastrointestinal standpoint and is also associated with a small, not-negligible, risk of bone marrow suppression [38]. In addition, some patients do not tolerate colchicine at therapeutic doses and up to 5% do not respond adequately to the highest tolerable dose: the TNF inhibitor etanercept has been ineffective in such patients, leading investigators to speculate the relative superiority of IL-1 blockade [39]. Indeed, IL-1 antagonists are the treatment of choice in refractory or colchicine-intolerant cases of FMF, and a large experience with anakinra and canakinumab is now available for thousands of colchicine-resistant patients [40]. Canakinumab seems to have a positive effect on different severe inflammatory symptoms by controlling disease activity and overall inflammation [41]. A better interpretation of FMF mutations in various clinical sceneries has led to coin the umbrella-term “pyrin-associated autoinflammatory diseases”, including all non-FMF diseases caused by *MEFV*-related pyrin defects, such as periodic undefined fevers, periodic fever/aphthosis/pharyngitis/adenitis syndrome-like pictures and neutrophilic dermatosis [42]. In 2019 Gattorno et al. have drafted the Eurofever/PRINTO classification criteria for CAPS, TRAPS, MKD and FMF [43] with the aim of identifying patients eligible for clinical, epidemiological or translational studies after excluding all other potential causes of fever, such as infections, neoplasms, autoimmune disorders and primary immunodeficiencies (see Table 3, Supplementary material).

Various pyogenic disorders starting in childhood can also be considered inflammasome-related diseases, and—particularly—a rare autosomal-dominant pathology caused by mutations in the *PSTPIP1* gene, named “pyogenic arthritis, pyoderma gangrenosum and acne (PAPA) syndrome”, is the most relevant [44]. The involved gene encodes the proline-serine-threonine phosphatase-interacting protein 1, a cytoskeleton-associated adaptor protein that binds pyrin and regulates IL-1 production; the syndrome is defined by self-limited episodes of oligoarthritides with an accumulation of neutrophil-rich, but sterile, material in the synovial fluid, severe pictures of pyoderma gangrenosum and disfiguring acne, which may evolve into necrotic plaques and atrophic scars [45]. The clinical management of PAPA syndrome can benefit from corticosteroids, TNF inhibitors, thalidomide and IL-1 antagonists [46]. A pyogenic autosomal-recessive disease caused by *LPIN2* mutations and leading to abnormal function of lipin-2, a phosphatidate phosphatase involved in the glycerolipid biosynthesis, is Majeed syndrome: this disease is defined by early-onset recurrent multifocal osteomyelitis, neutrophilic dermatosis and congenital dyserythropoietic anemia [47]. The role of lipin-2 as a key-participant in the regulation of proinflammatory gene expression by saturated fatty acids in macrophages has been recently clarified, and we also know that reduction of lipin-2 expression promotes a proinflammatory state in macrophages, generating more inflammatory cytokines with persistent inflammation [48]. Another pyogenic disease characterized by pustular rashes, aseptic multifocal osteomyelitis and increased acute-phase reactants in newborns identifies the deficiency of the naturally occurring competitive inhibitor of IL-1, which is the IL-1 receptor antagonist (a disease named “DIRA”), caused by *IL1RN* mutations: the absent inhibition of IL-1 in children with DIRA can be fully treated with the IL-1 receptor antagonist anakinra [49]. The diagnostic identification of such disorders relies on both clinical and radiological insights, but genetic testing of *PSTPIP1*, *LPIN2* and *IL1RN* is mandatory (the basic manifestations of inflammasomopathies with prominent pyogenic manifestations have been recorded in Table 4, Supplementary material).

## The relopathies

Nuclear factor-kappa light-chain enhancer of activated B cells (best known as NF-κB) represents a family of DNA transcription factors which coordinate a large array of genes involved in both inflammatory responses and infection-related immune processes, displaying powerful effects on cell differentiation and cell survival after stress. All proteins of the mammalian NF-κB family share a Rel homology domain in the N-terminus, and five proteins (NF-κB1, NF-κB2, RelA, RelB and c-Rel) can be found in almost all animal cell types if stimulated by free radicals, heavy metals, ultraviolet irradiation, bacterial or viral antigens [50]. NF-κB-related AIDs (recently denominated “relopathies”) encompass protean conditions, including Blau syndrome (BS) which is caused by autosomal dominant mutations in the *NOD2* gene and is characterized by recurrent granulomatous uveitis, boggy synovitis with symmetric camptodactyly and a peculiar scaly rash with ichthyosiform or lichenoid features [51]. The *NOD2* gene encodes the NACHT domain of the cytosolic multi-domain NOD2 (nucleotide-binding oligomerization domain containing 2), also known as caspase recruitment domain family member 15 (CARD15) protein, and mutant *NOD2* leads to upregulated NF-κB activation [52]. In particular, mutations in the *NOD2* gene are also found in children with early-onset sarcoidosis, a sporadically occurring condition that shares a common genetic etiology with BS. Histology of affected organs can reveal non-caseating granuloma in BS and its treatment relies on corticosteroids, immunosuppressant drugs, TNF inhibitors and IL-1 antagonists [53]. Sporadic or dominant gain-of-function mutations in the *CARD14* gene also activate NF-κB pathway and cause early-onset plaque psoriasis, pityriasis rubra pilaris and generalized pustular psoriasis, which have been named CARD14-mediated psoriasis (or CAMPS) [54]. Furthermore, homozygous loss-of-function mutations in the *IL36RN* gene cause the deficiency of the IL-36 receptor antagonist (also named DITRA) in keratinocytes, a rare disorder recognizable by severe generalized pustular psoriasis induced by upregulated NF-κB activity [55].

The overall NF-κB pathway is tightly controlled through multiple post-translational mechanisms, including protein modifications supervised by highly conserved ubiquitin peptides. Ubiquitylation is a three-step process performed by the concerted actions of ubiquitin-activating or ubiquitin-conjugating enzymes and hundreds of substrate-specific ligases. Ubiquitin-driven signaling is counterbalanced by deubiquitinase enzymes, such as OTULIN and A20: both mutant OTULIN-cells and mutant A20-cells display constitutive upregulation of NF-κB signaling [56]. Hypomorphic mutations in the *OTULIN* gene result in elevated NF-κB activity causing the OTULIN-related autoinflammatory syndrome (named also “otulipenia”), characterized by inflammatory skin signs and panniculitis which start in the neonatal period and respond to TNF inhibitors [57]. Similarly, patients with heterozygous mutations in the *A20* gene cause haploinsufficiency of A20 (also named HA20), displaying an excessive ubiquitination which leads to increased NF-κB activity and also NLRP3-inflammasome activation. HA20 patients present with some Behçet-like characteristics or an autoimmune lymphoproliferative syndrome-like phenotype [58]. Recently, using a genotype-driven approach, Beck et al. identified an autoinflammatory disorder related to somatic mutations in the *UBA1* gene, encoding the major enzyme that initiates ubiquitylation, which expresses itself in late adulthood with fevers, dysplastic bone marrow, cytopenia, vasculitis, skin and lung inflammatory signs [59]. Table 5 (Supplementary material) shows the most relevant manifestations of relopathies.

## Interferonopathies

Interferons (IFNs) have a variety of effects due to their strong antiviral, antitumor and immunomodulatory activities, and IFN-regulated genes (IRG) are upregulated in the blood, muscle and skin of patients with autoinflammatory interferonopathies, classically associated with a peculiar IRG signature, caused by dysregulation of type I IFNs (IFN-α, IFN-β, IFN-ω, IFN-ε and IFN-κ) activity. These diseases include proteasome-associated autoinflammatory syndromes (or PRAAS), caused by additive loss-of-function mutations in genes related to proteasome components (such as *PSMB8, PSMB9, PSMB7, PSMA3* or proteasome assembly factors as *POMP* and *PSMG2*), and STING-associated vasculopathy with onset during infancy (or SAVI), caused by gain-of-function mutations in the *TMEM173* gene, which encodes the stimulator of IFN genes (STING) protein, an evolutionarily conserved endoplasmic reticulum transmembrane protein with the downstream effect of activating IFN pathway [60]. Due to the pronounced IRG signature, blocking IFN with Janus kinase inhibitors (like tofacitinib or baricitinib) ensures clinical improvement in the majority of patients with interferonopathies [61]. In particular, the proteasome system is the most relevant pathway of intracellular degradation of proteins outside of lysosomes and is crucial for the preservation of cell viability in tissues exposed to different inflammatory noxae. PRAAS are a group of rare disorders (involving the best known “chronic atypical neutrophilic dermatosis with lipodystrophy and elevated temperature” or CANDLE syndrome), characterized by cell accumulation of ubiquitinated waste proteins that activate type I IFN signaling to drive inflammation. These patients display recurrent fevers, recurrent skin manifestations ranging from characteristic violaceous plaques with dermal mononuclear cell infiltrate to erythema nodosum-like panniculitis evolving to progressive lipodystrophy, muscle atrophy and small joint contractures [62]. Conversely, SAVI is a complex rare disorder characterized by neonatal-onset severe vasculitis localized to cheeks, ears, nose and fingers with risk of gangrene and chronic interstitial lung disease [63, 64]. As type I interferon pathway represents a relevant pathogenic mechanism for systemic lupus erythematosus and phenotypic overlapping with interferonopathies is possible, it is useful to remind that atypical or incomplete lupus-like symptoms occurring in infancy or in preprepubertal age should also recall the diagnosis of either PRAAS or SAVI. The distinctive manifestations which characterize interferon-related AIDs are listed in Table 6 (Supplementary material).

## Non-hereditary autoinflammatory disorders

**Fig. 4:**
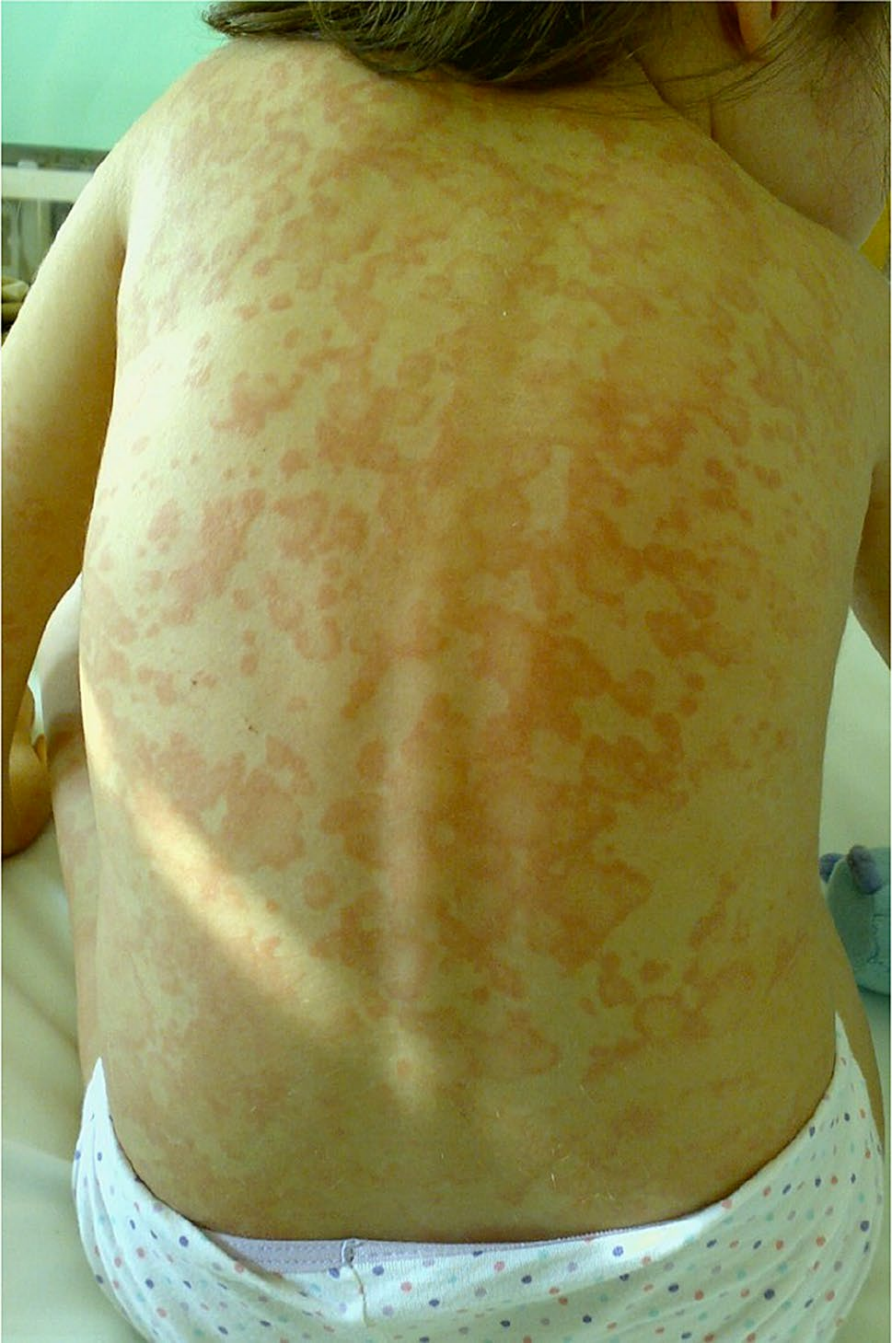
A typical short-lived evanescent nonfixed evanescent salmon-pink rash that appears with fever peaks and fades away when the fever subsides in a child with systemic juvenile idiopathic arthritis

In addition to hereditary monogenic AIDs, other syndromes encountered in children have been included in the group of multifactorial AIDs with a presumed polygenic basis. Furthermore, the description of unusual phenotypes of AIDs with some autoimmune features has also highlighted some potential links between innate and adaptive immune responses. In particular, systemic juvenile idiopathic arthritis (SJIA), included in the group of juvenile arthritides, has peculiar characteristics deriving from uncontrolled activation of phagocytes with hypersecretion of IL-1 and IL-6, which are opposite in comparison with the other forms of juvenile idiopathic arthritis, the most common chronic rheumatic disease of childhood, i.e. prominent joint involvement, strong association with some HLA class II antigens and eventual positivity of anti-nuclear antibodies, rheumatoid factor or anti-cyclic citrullinated peptide antibodies [65]. SJIA is defined by the presence of spiking fevers in the child, which persist for a minimum of two weeks, combined with at least one among evanescent nonfixed skin rash (Fig. 4), generalized lymphadenopathy, hepatosplenomegaly or serositis [66]. Table 7 (in the Supplementary material) shows the definition of SJIA according to the recent 2019 PRINTO criteria.

None of the clinical signs included as major or minor criteria is specific to SJIA, especially at presentation, and fatal complications may also occur, such as macrophage activation syndrome, a form of reactive hemophagocytic lymphohistiocytosis characterized by uncontrolled activation of well-differentiated macrophages releasing proinflammatory cytokines [67]. Based on clinical features as well as on the new acquisitions about SJIA pathogenesis showing IL-1 dysregulation, it is justified that this disorder being included in the group of non-hereditary AIDs, and this hypothesis is also confirmed by the paramount response to anti-IL-1 agents seen in SJIA, though further data are needed to explain whether this is due to intrinsic abnormalities of caspase-1 pathway [68].

A more frequent cause of recurrent fevers in children is periodic fever/aphthosis/pharyngitis/adenitis (PFAPA) syndrome, defined by febrile attacks having “clockwork” periodism accompanied by stereotyped symptoms located at the oral cavity and neck alternating with periods of complete well-being: this is a benign disease with a remarkable impact on child’s and parents’ quality of life, though its outcome is generally favorable with spontaneous remission after a variable number of years [69]. The syndrome is largely observed in children less than 6 years showing recurring fevers every 3–6 weeks combined with at least one among aphthous stomatitis, pharyngitis and/or cervical lymph node enlargement with no evidence of upper airways infections [70]. There are limited studies focusing on the infradian cycle of PFAPA symptom recurrence, though clock-related genes and their relationship with immunity have been recognized [71]. Diagnosis of PFAPA syndrome in a pediatric scenery requires laboratory evaluations both during and between febrile episodes, detailed history-taking and mindful collection of all physical findings to exclude primary immunodeficiency disorders or hereditary AIDs. This is important as low-dose corticosteroids can brilliantly relieve most PFAPA symptoms presented by children, while colchicine may add some help in those patients carrying heterozygous *MEFV* gene mutations [72]. An IL-1-mediated pathogenesis suggests the autoinflammatory origin of PFAPA syndrome, which appears as a rhythmic self-limited défaillance in the regulation of innate immunity, disrupting the oral ecosystem and camouflaging the recognition of resident commensal microbial communities in the tonsils [73]. A delayed onset of PFAPA syndrome during adulthood has been described as well, though relevant differences in response to treatment were observed between children and adults [74, 75]. Table 8 (in the Supplentary material) shows the definition of PFAPA syndrome in childhood according to Marhall’s criteria and according to Eurofever/PRINTO classification criteria.

**Fig. 5:**
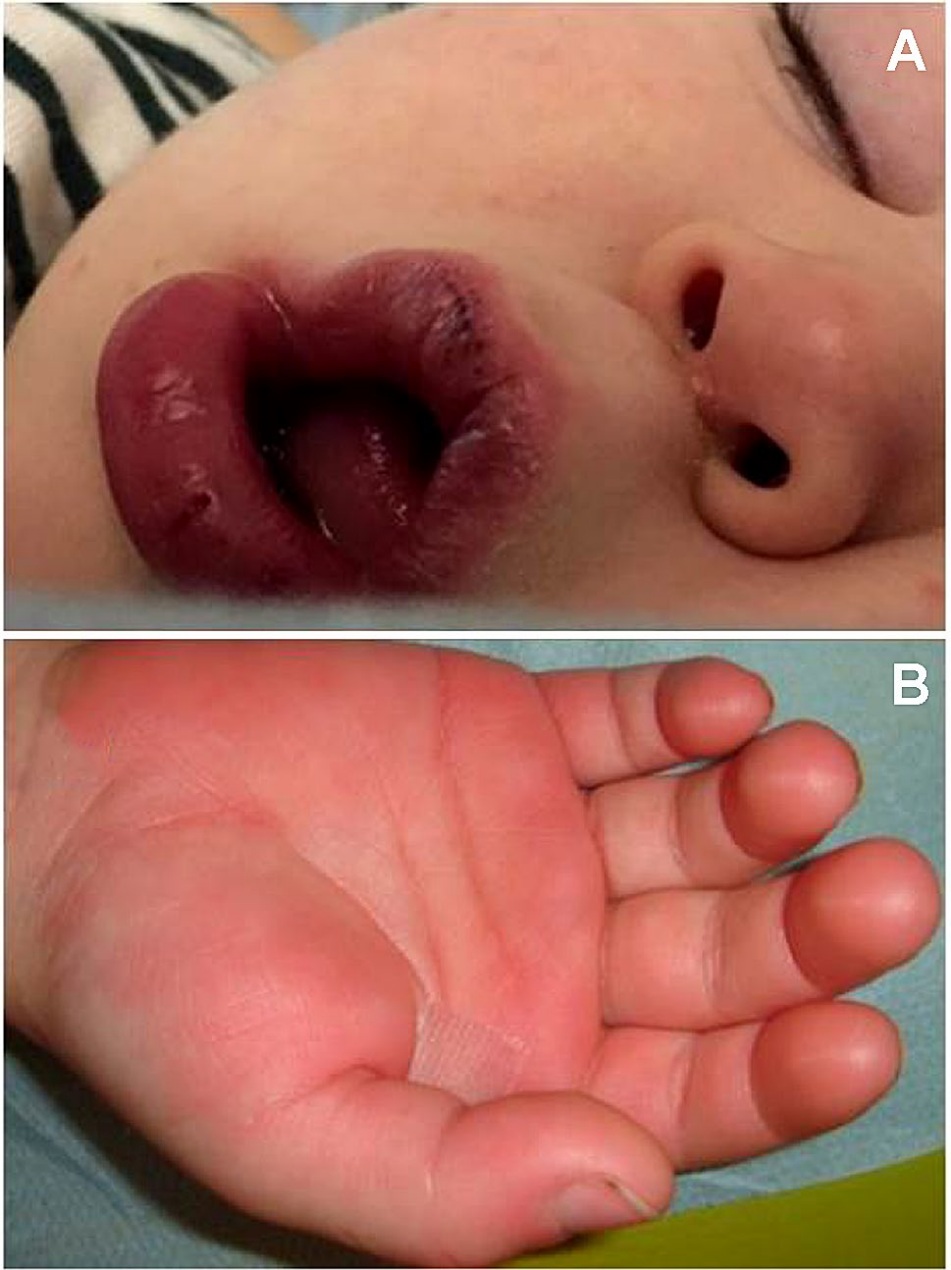
A typical mouth with swollen lips/vertical cracking **a** and a hand with palm erythema/indurative edema **b** in a child diagnosed with Kawasaki disease

Another typical febrile disorder with a presumed autoinflammatory basis is Kawasaki disease (KD), an acute self-limiting vasculitis of unknown etiology which has been mostly recognized in children of Asian descent, characterized by persistently high and unremitting fever combined with nonspecific skin and facial signs (Table 9 in the Supplementary material lists the classic manifestations required for the diagnosis of KD) [76]. Figure 5 shows the details of mouth and hand in a child with KD. This enigmatic illness might damage the coronary arteries in a quarter of untreated patients, making KD the most common cause of childhood-acquired heart disease in the developed countries: the underlying etiology and mechanisms which provoke vessel inflammation, coronary artery lesions and aneurysms that are hallmarks of KD remain unknown. The risk of cardiovascular mortality is highest in the first year after KD, but myocardial infarctions have been also increasingly reported in young adults with a missed diagnosis of KD [77]. Treatment with intravenously administered immunoglobulin has the goal of reducing inflammation in the coronary arteries and myocardium during the acute phase of KD, and nonresponders to intravenous immunoglobulin are those who will probably develop coronary artery abnormalities [78]. A better understanding of the vascular pathology should help identifying targeted therapies and preventing the cardiac complications of KD: however—despite more than 5 decades of investigation—many scientific gaps still exist and troubling areas of debate include a genetic predisposition to the disease involving autoinflammatory pathways and non-responsiveness to intravenous immunoglobulin in a subset of KD cases [79]. A host of studies indicates that KD primum movens may be a dysregulated immune response to various microbial agents, causing both cytokine overproduction and endothelial cell dysfunction at least in the genetically predisposed patients who develop the disease [80, 81]. To date, several infectious agents have been proposed as potential triggers of KD, but none has been consistently demonstrated as the unique culprit; conversely, the role of different susceptibility genes predisposing to KD remains unraveled. Concurrently, the upregulation of the innate immune system combined with relative abundance of autoinflammation-related gene transcripts, a high number of circulating neutrophils and hypersecretion of IL-1, IL-6 and TNF as seminal events in patients with KD explain the usefulness of anti-cytokine treatments in the refractory forms [82]. In particular, IL-1α triggers the inflammatory response in the initial phase of KD and likely regulates IL-1β secretion, whose role dominates the later disease phases [83]. Caspase-1 and IL-1α have been also shown to play a role in the development of CAA, warranting the successful use of the IL-1 blocker anakinra, acting on both IL-1α and IL-1β, for the coronary artery abnormalities of KD [84].

## Conclusion

Health-care workers called to evaluate the manifold potential causes of recurrent fevers in children and adolescents should contemplate AIDs, a growing family of rare chronic syndromes characterized by early-onset, neonatal or in the first years of life, and lifelong recurrent and seemingly unprovoked inflammatory episodes caused by innate immunity activation in the absence of any recognizable pathogen. These diseases have been recently framed as pleiotropic cytokinopathies, due to the fact that specific cytokine signaling may have a dominating contribution to define the pathology and become an ideal target of customized therapies [85]. Advances in the understanding of inflammasome assembly and properties have potentiated the use of novel therapeutics against inflammasome-related diseases, but a similar success has been also observed for both relopathies and interferonopathies.

In general terms, a dysregulation of innate immunity leading to an intense production of proinflammatory cytokines is central to the pathogenesis of AIDs. Investigating family recurrence of fevers, ethnicity, early-onset fevers within the first span of life, duration of febrile episodes as well as length of intervals between episodes and concomitant recurrence of organ-specific manifestations might suggest an innate immunity defect. This review has sketched the phenotypic diversity of AIDs occurring in children, which—apart from fever—can be sometimes complicated by the occurrence of dramatic life-threatening pictures, such as macrophage activation syndrome which is burdened by dramatically high rates of mortality [86]. Further progress in molecular sequencing has allowed a stricter genetic characterization of AIDs (see Table 10 in the Supplementary material), and key points for a proper identification of AIDs have been shown in Box 1.

Box 1 Key-points for the identification of hereditary monogenic autoinflammatory disorders

**Table:** 

*History details suggesting an autoinflammatory disorder*
Recurrent febrile attacks with localized symptoms (abdominal pain, arthro-myalgia, self-limited arthritis, skin rash, etc.)
Recurrent acute serositis (peritonitis, pleurisy, pericarditis)
Family history of similar attacks in horizontal or vertical distribution
*Factors discriminating among hereditary monogenic autoinflammatory disorders*
Peculiar ethnic origin (at least for patients with familial Mediterranean fever)
Mode of inheritance
Overall duration of each febrile/inflammatory attack
Response to colchicine or to corticosteroids
Presence of a confirmatory result at the genetic test
*Specific clues indicating the diagnosis of a monogenic autoinflammatory disorder*
Erysipelas-like erythema on foot/ankle and response to long-term colchicine prophylaxis (familial Mediterranean fever)
Longer duration of febrile attacks with migratory myalgia, periorbital edema, conjunctivitis and corticosteroid-induced suppression of fever (autosomal dominant familial periodic fever, also known as tumor necrosis factor-associated periodic syndrome)
Onset in the first year of life with febrile attacks (often triggered by vaccinations) characterized by recurring intestinal/cutaneous signs, lymph node enlargement and splenomegaly (mevalonate kinase deficiency, also known as hyper-IgD syndrome)
Urticaria-like rash, neurosensorial hypoacusia, osteo-articular involvement of variable severity, neurological signs and circadian periodicity of symptoms (cryopyrin-associated periodic syndrome)
Acute “pyogenic” manifestations involving bone, joints and skin (hereditary pyogenic disorders
Granulomatous polyarthritis with uveitis and ichthyosiform rash (pediatric granulomatous arthritis, i.e. Blau syndrome and early-onset sarcoidosis)
In vivo*-test to evaluate the predominating cytokine involved in hereditary monogenic autoinflammatory disorders*
Administration of anakinra (as interleukin-1-mediated disorders display a brilliant response to anakinra)

Obtaining a family history is an essential part of the evaluation protocol for such patients: priority is to examine child’s clinical and laboratory data both in the acute inflammatory phase and in the intercritical phase, to confirm the presence of subclinical inflammation between attacks and exclude a host of chronic diseases of infectious, autoimmune or neoplastic nature as well as immunodeficiencies. An additional contribution to diagnosis derives from the consideration of ethnicity and genotype analysis, but a thorough differential diagnosis results from integrating clinical data and “focused” genetic tests [87]. Specific supportive tools to measure chronic damage caused by AIDs and define prognosis have been developed for children and adults [88] or are currently in progress [89]. As we improve our understanding of the complexity of innate immunity, novel adaptations may lend support to the ultimate development of experimental and computational tools to fully capture the mechanics of immune responses in healthy subjects and show how they may be leveraged to fight AIDs or potentially improve the long-term prognosis of these diseases.

## Supplementary Information

Below is the link to the electronic supplementary material.

Supplementary file1 (DOC 155 KB)

## Abbreviations

BS: Blau syndrome
CAPS: Cryopyrin-associated periodic syndrome
DIRA: Deficiency of the interleukin-1 receptor antagonist
FMF: Familial mediterranean fever
MKD: Mevalonate kinase deficiency
MS: Majeed syndrome
PAPAs: Pyogenic arthritis, pyoderma gangrenosum and acne syndrome
PRAAS: Proteasome-associated autoinflammatory syndromes
TRAPS: Autosomal dominant familial periodic fever (tumor necrosis factor receptor-associated periodic syndrome
