# Supplementary material for: The everchanging framework of autoinflammation
Source: Intern Emerg Med. 2021 May 17;16(7):1759–70. doi: 10.1007/s11739-021-02751-7 (PMC8502124; doi:10.1007/s11739-021-02751-7)
Supplement: Supplementary file 1 — Supplementary file1 (DOC 155 KB) [file 11739_2021_2751_MOESM1_ESM.doc]

SUPPLEMENTARY MATERIAL

**The everchanging framework of autoinflammation**

Raffaele Manna^1,2^ & Donato Rigante^3,2^

^1^Department of Internal Medicine, Fondazione Policlinico A. Gemelli IRCCS, Rome, Italy.

^2^Rare Diseases and Periodic Fevers Research Centre, Università Cattolica Sacro Cuore, Rome, Italy.

^3^Department of Life Sciences and Public Health, Fondazione Policlinico A. Gemelli IRCCS, Rome, Italy.

**Table 1.** Summary of the clinical phenotypes observed in cryopyrin-associated periodic syndrome, differentiated in familial cold autoinflammatory syndrome, Muckle-Wells syndrome and CINCA syndrome.

|  | ***Familial cold autoinflammatory syndrome*** | ***Muckle-Wells***  ***syndrome*** | ***Chronic infantile neurologic cutaneous articular*** *(****CINCA) syndrome*** |
| --- | --- | --- | --- |
| *Onset age* | First infancy | Childhood | Neonatal (or prenatal) period |
| *Duration of inflammatory signs* | Usually short | Daily or subcontinuous | Continuous with exacerbations |
| *Skin manifestations* | Urticaria-like rash a few hours after generalized cold exposure (an ice cube on the skin does not usually provoke the rash) | Urticaria-like rash with variable exacerbations, localized erythema around the nails and over the knuckles (mimicking Gottron papules) | Early-onset urticaria-like rash, persistent migrating polymorphous rash |
| *Musculo-skeletal symptoms* | Arthralgia, joint stiffness | Lifelong arthralgias,  non-erosive polyarthritides, nail clubbing | Deforming osteo-arthropathy of large joints, monstrous epiphyseal overgrowth (mimicking a “bone mass”), abnormal premature ossification of the patella, joint contractures, digital clubbing |
| *Audiologic assessment* | Usually normal | Sensorineural hearing loss (for high-pitched sounds) | Sensorineural hearing loss |
| *Ocular signs* | Recurring mild conjunctivitis | Conjunctivitis | Chronic papilledema, optic nerve atrophy, visual loss, uveitis |
| *Neurologic signs* | - | Transient drowsiness | Chronic aseptic meningitis |
| *Systemic signs and laboratory abnormalities* | Fever spikes of short duration, profuse sweating (after cold exposure), thirst | Recurrent bouts of fever | Recurrent fevers with shivers or persistent mild fever, constant elevation of inflammatory markers, neutrophil leukocytosis |
| *Dysmorphic features* | - | - | Frontal bossing, saddle nose, midface hypoplasia |
| *Long-term sequelae* | Fatigue | Fatigue, amyloidosis | Amyloidosis |

**Table 2.** Classification criteria for the diagnosis of familial Mediterranean fever. According to Tel Hashomer criteria (1967) diagnosis requires 2 major criteria or 1 major and 2 minor criteria satisfied, while diagnosis is probable if 1 major and 1 minor criteria are present. According to Livneh’s criteria (1997) diagnosis requires ≥1 major criteria, or ≥2 minor criteria, or 1 minor criterion plus ≥5 supportive criteria, or 1 minor criterion plus ≥ 4 of the “first” 5 supportive criteria in the list.

| **Tel Hashomer criteria** | **Livneh’s criteria** |
| --- | --- |
| **Major** | **Major** |
| Recurrent fevers + peritonitis/synovitis/pleurisy | Typical attack of peritonitis |
| AA amyloidosis | Typical attack of unilateral pleuritic or pericarditis |
| Favorable response to colchicine | Typical attack of monoarthritis |
| **Minor** | Fever (rectal temperature of 38°C or higher) alone |
| Recurrent fevers | **Minor** |
| Erysipelas-like erythema | Incomplete attack involving the abdomen |
| Family history of FMF in a first-degree relative | Incomplete attack involving the chest |
|  | Incomplete attack involving one large joint |
|  | Exertional leg pain |
|  | Favorable response to colchicine |
|  | **Supportive** |
|  | Family history of familial Mediterranean fever |
|  | Typical ethnic origin (Armenian, Turkish, Arabian, Hispanic Jew) |
|  | Age less than 20 years at disease onset |
|  | Severity of attack requiring bed rest |
|  | Spontaneous remission of attacks |
|  | Symptom-free intervals between attacks |
|  | Transient increase of inflammatory parameters during attacks |
|  | Episodic proteinuria or hematuria |
|  | Surgical removal of a “white” appendix |
|  | Consanguinity of parents |

**Table 3.** Eurofever/PRINTO classification criteria for the main four inflammasomopathies spread in 2019 for identifying patients with fevers recurring in a period of at least 6 months (combined with elevation of acute-phase reactants), who are candidates for experimental studies.

| ***Cryopyrin-associated periodic syndrome*** | ***Autosomal dominant familial periodic fever*** *(tumor necrosis factor receptor-associated periodic syndrome)* | ***Mevalonate kinase deficiency*** | ***Familial Mediterranean fever*** |
| --- | --- | --- | --- |
| Presence of a confirmatory *NLRP3* genotype and at least 1 among:  - Urticaria-like rash  - Eye inflammation  - Sensorineural hearing loss  or  Presence of a non-confirmatory *NLRP3* genotype and at least 2 among:  - Urticaria-like rash  - Eye inflammation  - Sensorineural hearing loss | Presence of a confirmatory *TNFRSF1A* genotype and at least 1 among:  - Attacks lasting ≥7 days  - Myalgia  - Migratory skin rash  - Positive family history  or  Presence of a non-confirmatory *TNFRSF1A* genotype and at least 2 among:  - Attacks lasting ≥7 days  - Myalgia  - Migratory skin rash  - Positive family history | Presence of a confirmatory *MVK* genotype and at least 1 among:  - Gastrointestinal symptoms  - Cervical lymphadenopathy  - Aphthous stomatitis | Presence of a confirmatory *MEFV* genotype and at least 1 among:  - Attacks lasting 1-3 days  - Arthritis  - Chest pain  - Abdominal pain  or  Presence of a non-confirmatory *MEFV* genotype and at least 2 among:  - Attacks lasting 1-3 days  - Arthritis  - Chest pain  - Abdominal pain |

**Table 4. General details and onset times of inflammasome-related “pyogenic” autoinflammatory disorders with onset in childhood.**

|  | *Onset* | *Clinical signs* |
| --- | --- | --- |
| **PAPA syndrome** | First infancy | Skin ulcerations, pyoderma gangrenosum, cystic acne,  sterile pyogenic oligoarthritides |
| **Majeed syndrome** | First 2 years of life | Diffuse neutrophilic dermatosis, chronic non-bacterial osteomyelitis, dyserythropoietic anemia |
| **Deficiency of the interleukin-1 receptor antagonist** | Neonatal period (or prenatal period) | Pustular rash, ichthyosis-like changes of the skin, multifocal osteomyelitis, nail abnormalities, risk of multi-organ failure |

**Table 5. General details and onset times of relopathies starting in childhood.**

|  | *Onset* | *Clinical signs* |
| --- | --- | --- |
| **Blau syndrome** | First infancy | Brown-coloured scaly and ichthyosiform rash or lichenoid rash, recurrent polyarthritis, granulomatous uveitis (anterior, posterior or intermediate), risk of ocular sequelae (synechiae, cataracts, band keratopathy) |
| **CARD14-mediated psoriasis** | Variable (many cases may start in the first infancy) | Plaque psoriasis, pityriasis rubra pilaris, pustular psoriasis, joint pain, recurrent fevers |
| **Deficiency of the interleukin-36 receptor antagonist** | Variable (many cases may start in the first infancy) | Generalized severe pustular psoriasis, acute generalized exanthematous pustulosis, palmar-plantar pustulosis, acrodermatitis continua of Hallopeau, disseminated subcorneal pustules, recurrent fevers, asthenia, fatigue |
| **OTULIN-related autoinflammatory syndrome** (otulipenia) | First infancy | Recurrent fevers, erythematous skin rash with nodules, arthralgia, abdominal pain, diarrhea, lymphadenopathy |
| **A20 haploinsufficiency** | First or second decade | Recurrent fevers, early-onset manifestations resembling Behçet’s disease, early-onset manifestations resembling autoimmune lymphoproliferative syndrome, aphthous stomatitis, oral and genital ulcers, uveitis, polyarthritis |

**Table 6.** General details and onset times of interferon-related autoinflammatory disorders starting in childhood.

|  | *Onset* | *Clinical signs* |
| --- | --- | --- |
| **Proteasome-associated autoinflammatory syndromes** | Early childhood | Recurrent fevers, pernio-like skin rash, annular erythematous plaques on the face and extremities with subsequent development of panniculitis-induced lipodystrophy (loss of adipose tissue), severe joint contractures, large eyes, nose, lips and ears, eyelid swelling, uveitis, disproportionately long and thick fingers, muscle weakness and atrophy, hepatosplenomegaly, basal ganglia calcifications, microcytic anemia, mild mental retardation |
| ***STING*-associated vasculopathy with onset in infancy** | Neonatal period | Severe vasculitis, violaceous scaling lesions on fingers, toes or nose (which are exacerbated by cold exposure) that might progress to chronic ulceration of extremities, autoamputation phenomena, chronic interstitial lung disease, failure to thrive |

**Table 7.** Clinical definition of systemic juvenile idiopathic arthritis (according to the 2019 PRINTO criteria): diagnosis requires that the cardinal sign is associated with 2 major criteria or with 1 major criterion and 2 minor criteria, after exclusion of infectious, neoplastic, autoimmune and autoinflammatory diseases.

| *Cardinal sign* | *Major criteria* | *Minor criteria* |
| --- | --- | --- |
| Fever of unknown origin that is documented to be daily (until 39°C once a day with intermittent course) for at least 3 consecutive days and reoccurring over a duration of at least two weeks | 1. evanescent nonfixed erythematous rash 2. arthritis | 1. generalized lymph node enlargement and/or hepatomegaly and/or splenomegaly 2. serositis 3. arthralgia lasting 2 weeks or longer (in the absence of arthritis) 4. leukocytosis (≥15.000/mm^3^) with neutrophilia |

**Table 8.** Clinical definition of PFAPA syndrome in childhood.

| **Marshall’s criteria** | *Basic signs* | *Location signs* | *Exclusion criteria* |
| --- | --- | --- | --- |
|  | - Periodically recurring fever at almost precise intervals of about 3-6 weeks - Child’s complete well-being between flares with normal growth and development  - Association with at least 1 location sign | 1. aphthous stomatitis 2. pharyngitis 3. cervical lymph node enlargement | - Upper respiratory airway infections - Primary immunodeficiency disorders - Hereditary autoinflammatory diseases - Cyclic neutropenia |
| **Gattorno’s criteria** | At least 7 out of 8: | *Presence of* | *Absence of* |
|  |  | 1. pharyngotonsillitis 2. febrile flares lasting 3-6 days 3. cervical lymphadenitis 4. “periodic” recurrence of flares | 1. diarrhea 2. chest pain 3. skin rash 4. arthritis |

**Table 9.** Clinical features useful to recognize and diagnose Kawasaki disease.

| Fever persisting at least for 5 days (or more) *plus* at least 4 of the following 5 clinical signs: |
| --- |
| 1. Bilateral bulbar conjunctival injection without exudate |
| 1. Polymorphous skin rash |
| 1. Changes in lips (reddened, cracked or dry) and oral cavity (strawberry tongue, diffuse oral and pharyngeal hyperemia) |
| 1. Changes in the extremities and in the perineum (erythema of palms or soles, indurative edema of hands or feet, desquamation of perineal skin) |
| 1. Acute cervical lymph node enlargement (>15 mm) |

**Table 10.** Classification of the hereditary [autoinflammatory disorders](https://www.sciencedirect.com/topics/medicine-and-dentistry/autoinflammatory-disease) with genes involved and therapies depending on [signaling pathway](https://www.sciencedirect.com/topics/medicine-and-dentistry/signal-transduction)s engaged in each disorder.

| Disorder | Gene | Protein encoded | Inheritance | Treatment choices |
| --- | --- | --- | --- | --- |
| **Cryopyrin-associated periodic syndrome** (CAPS) | NLRP3 | Cryopyrin (NLRP3) | autosomal dominant | Interleukin-1 antagonists (canakinumab, anakinra) |
| **Autosomal dominant familial periodic fever**  (Tumor necrosis factor receptor-associated periodic syndrome) | TNFRSF1A | p55 tumor necrosis factor receptor | autosomal dominant | Corticosteroids, canakinumab |
| **Mevalonate kinase deficiency**  (Hyper-IgD syndrome) | MVK | Mevalonate kinase | autosomal recessive | Nonsteroidal anti-inflammatory drugs,  canakinumab, anakinra “on demand” |
| **Familial Mediterranean fever** | MEFV | Pyrin (TRIM20 or marenostrin) | autosomal recessive | Colchicine, canakinumab, anakinra |
| **PAPA syndrome** | PSTPIP1 | CD_2_ antigen-binding protein 1 | autosomal dominant | Corticosteroids, TNF-α inhibitors, anakinra,  canakinumab, cyclosporine, tacrolimus,  thalidomide |
| **Majeed syndrome** | LPIN2 | Lipin-2 | autosomal recessive | Nonsteroidal anti-inflammatory drugs,  anakinra, corticosteroids, bisphosphonates,  TNF-α inhibitors, red blood cell transfusions |
| **Deficiency of the interleukin-1 receptor antagonist** (DIRA) | IL1RN | Interleukin-1 receptor antagonist | autosomal recessive | Anakinra |
| **Blau syndrome, early-onset sarcoidosis** | NOD2/CARD15 | NOD2/CARD15 | autosomal dominant | Corticosteroids, TNF-α inhibitors, IL-1 antagonists |
| **CARD14-mediated psoriasis** (CAMPS) | CARD14 | CARD14 | autosomal dominant | Ustekinumab, ixekizumab |
| **Deficiency of the IL-36 receptor antagonist** (DITRA) | IL36RN | Interleukin-36 receptor antagonist | autosomal recessive | Corticosteroids, cyclosporine, acitretin,  adalimumab, anakinra |
| **OTULIN-related autoinflammatory syndrome** (Otulipenia) | OTULIN | OTULIN (deubiquitinase) | autosomal recessive | TNF-α inhibitors |
| **Haploinsufficiency of A20** | TNFAIP3 (A20) | A20 (deubiquitinase) | autosomal dominant | TNF-α inhibitors |
| [**Proteasome**](https://www.sciencedirect.com/topics/medicine-and-dentistry/proteasome)**-associated autoinflammatory syndromes** (PRAAS)- CANDLE syndrome | PSMB8, PSMB9, PSMB7, PSMA3, POMP, PSMG2 | Proteasome subunits or proteasome assembly factors | autosomal recessive | Corticosteroids, immunosuppressant agents, anakinra, tocilizumab, TNF-α  inhibitors, Janus kinase inhibitors |
| **STING-associated vasculopathy with onset in infancy** (SAVI) | TMEM173 | STING protein | autosomal dominant | Janus kinase inhibitors |
